# Genomic Characterization of Phenylalanine Ammonia Lyase Gene in Buckwheat

**DOI:** 10.1371/journal.pone.0151187

**Published:** 2016-03-18

**Authors:** Karthikeyan Thiyagarajan, Fabio Vitali, Valentina Tolaini, Patrizia Galeffi, Cristina Cantale, Prashant Vikram, Sukhwinder Singh, Patrizia De Rossi, Chiara Nobili, Silvia Procacci, Antonella Del Fiore, Alessandro Antonini, Ombretta Presenti, Andrea Brunori

**Affiliations:** 1 Italian National Agency for New Technologies, Energy and Sustainable Economic Development (ENEA), UTAGRI- INN, Via Anguillarese, 301, 00123 Rome, Italy; 2 Bioversity International, 00054, Maccarese (Fiumicino), Italy; 3 Genetic Resource Program, International Center for Maize and Wheat Improvement, El Batan, Texcoco, Mexico; National Institute of Plant Genome Research, INDIA

## Abstract

Phenylalanine Ammonia Lyase (PAL) gene which plays a key role in bio-synthesis of medicinally important compounds, Rutin/quercetin was sequence characterized for its efficient genomics application. These compounds possessing anti-diabetic and anti-cancer properties and are predominantly produced by *Fagopyrum* spp. In the present study, PAL gene was sequenced from three *Fagopyrum* spp. (*F*. *tataricum*, *F*. *esculentum* and *F*. *dibotrys)* and showed the presence of three SNPs and four insertion/deletions at intra and inter specific level. Among them, the potential SNP (position 949^th^ bp G>C) with Parsimony Informative Site was selected and successfully utilised to individuate the zygosity/allelic variation of 16 *F*. *tataricum* varieties. Insertion mutations were identified in coding region, which resulted the change of a stretch of 39 amino acids on the putative protein. Our Study revealed that autogamous species (*F*. *tataricum)* has lower frequency of observed SNPs as compared to allogamous species (*F*. *dibotrys* and *F*. *esculentum*). The identified SNPs in *F*. *tataricum* didn’t result to amino acid change, while in other two species it caused both conservative and non-conservative variations. Consistent pattern of SNPs across the species revealed their phylogenetic importance. We found two groups of *F*. *tataricum* and one of them was closely related with *F*. *dibotrys*. Sequence characterization information of PAL gene reported in present investigation can be utilized in genetic improvement of buckwheat in reference to its medicinal value.

## Introduction

**Fig 1:**
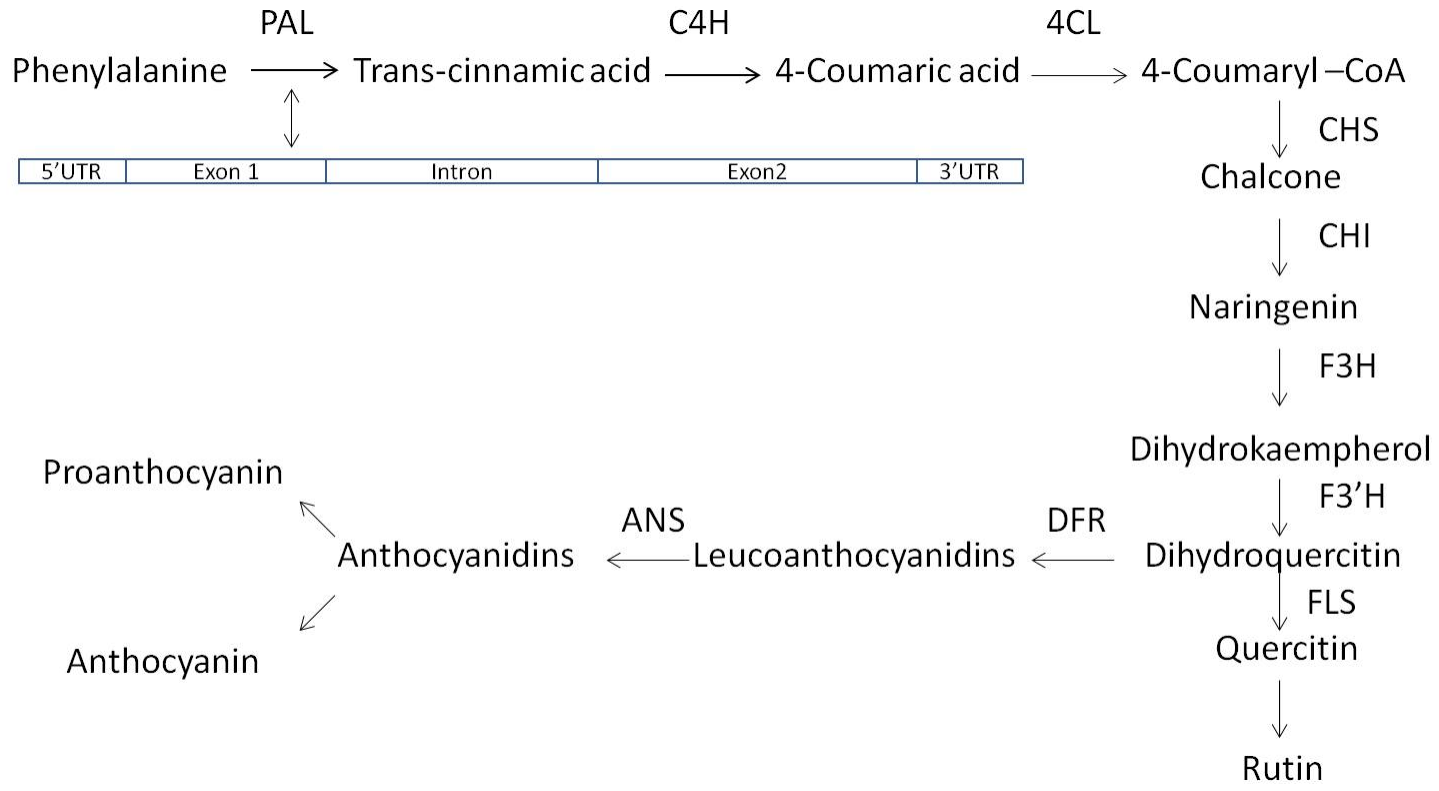
Description of PAL gene and rutin synthesis pathway. PAL, Phenylalanine Ammonia Lyase; C4H, Cinnamate 4-Hydroxylase; 4CL1 & 2, 4-Coumaroyl CoA Ligase; CHS, Chalcone Synthase; CHI, Chalcone Isomerase; F3H, Flavone 3-Hydroxylase; F3′H, Flavonoid 3′-Hydroxylase; FLS1&2, Flavonol Synthase; ANS, Anthocyanidin Synthase; Source [8].

Rutin and Quercetin are plant metabolites having antioxidant property and play a significant role in combating diabetes [1]. Diabetes is a chronic metabolic disorder resulted in mortality of over one million people globally [2]. Besides diabetes, rutin helps in reducing severity of colon carcinogenesis [3] and hypertension [4]. Rutin is neither present in cereals nor in pseudocereals [5]. Buckwheat is the only field crop species which possess rutin in the form of its secondary metabolite product [6]. Buckwheat generally grows at high altitude mountainous area, which derives the evolutionary mechanism of protecting itself against UV rays [7]. Rutin and quercetin are synthesized by a cascade of enzymes, in which Phenylalanine Ammonia Lyase (PAL; E.C 4.3.1.5) gene (PAL gene) is the first enzyme, which catalyses the conversion of precursor amino acid ‘phenylalanine’ to ‘trans-cinnamic acid’. Subsequently, several cascade of enzymes catalyzes further on each substrates until dihydroquercetin and finally Flavonol Synthase (FLS) converts dihydroquercetin to quercetin and rutin [8] (Fig 1).

*F*. *tataricum*, a pseudo cereal commonly known as ‘tartary buckwheat’ is rich in rutin [9]. During the culinary preparations, rutin (flavonol 3-O-rutinoside) is hydrolysed to a bitter compound called, quercetin, which gives strong bitter taste [10]. It is therefore very crucial to analyse *Fagopyrum* spp. with reference to genes involving in rutin and quercetin production. PAL gene is an important candidate involves in rutin/quercetin production and is studied extensively in this report. Tatary buckwheat contains 40 mg/g of flavonoids compared to common buckwheat (*F*. *esculentum* with 10 mg/g), among which rutin occupies a major portion [11]. SNPs are present in plant genomes at a high frequency and can be utilized efficiently as molecular markers for complex trait [12]. Allelic characterization of PAL gene is an important strategy for the genetic improvement of *Fagopyrum* spp. concerning the enhancement of rutin and quercetin content. PAL gene sequence information of *F*. *tataricum* (2864bp) and *F*. *dibotrys* (2583 bp) is well known [13] but, variation at single nucleotide polymorphism (SNP) level have not been reported yet. SNP variation in PAL gene can be identified in different accessions of the *Fagopyrum* spp. for their deployment in genetic improvement program. Amplification of the gene to identify SNPs can be used through cost effective strategies like Tetra primer ARMS PCR [14] for allele mining.

The genus *Fagopyrum* belongs to the family, Polygonaceae and consists of about 16 species [15] including common buckwheat (*F*. *esculentum*), tartary buckwheat and wild perennial buckwheat (*F*. *cymosum* or *F*. *dibotrys*). Among these two species, *F*. *tartaricum* and *F*. *esculentum* are domesticated. *F*. *esculentum* is the most commonly used species for human consumption, so called, ‘common buckwheat’ or ‘sweet buckwheat’ followed by *F*. *tataricum*, which is comparatively bitter in taste (therefore, also known as ‘bitter buckwheat’).

*F*. *esculentum* is widely grown in the temperate regions of Eurasia and North America, while the cultivation of tartary buckwheat is confined to Himalayan hills and some parts of Southern China. The latter areas are recognised as the natural habitat of the *Fagopyrum* genus including its wild relatives [16]. PAL gene has not been studied in most wild relatives of *Fagopyrum* genus. However, since past few decades research efforts have been given on *F*. *dibotrys*, led to utilize this species extensively for the characterization of this gene [11]. Rutin is a phenolic compound present in high concentrations in ‘tartary buckwheat’ and to limited extent in ‘common buckwheat’ [17]. Very little information is available concerning the genetic analysis of different species of the genus *Fagopyrum*, which led this genus remain underutilized. *F*. *esculentum* and *F*. *dibotrys* are allogamous, whereas *F*. *tartaricum* is an autogamous species. Morphological similarities suggested a greater closeness between *F*. *dibotrys* and *F*. *esculentum* [18, 19]. Conversely, recent RFLP-cpDNA molecular analysis revealed that *F*. *dibotrys* is more closely related to *F*. *tataricum* compared to *F*. *esculentum* [20]. An in-depth characterization of different *Fagopyrum* species with important genes (such as PAL gene) will lead to an increased taxonomic understanding and ultimately helps in their genetic enhancement as a crop of economic value.

## Results

### Allele mining of PAL gene in *F*. *tataricum* and related species

**Fig 2:**
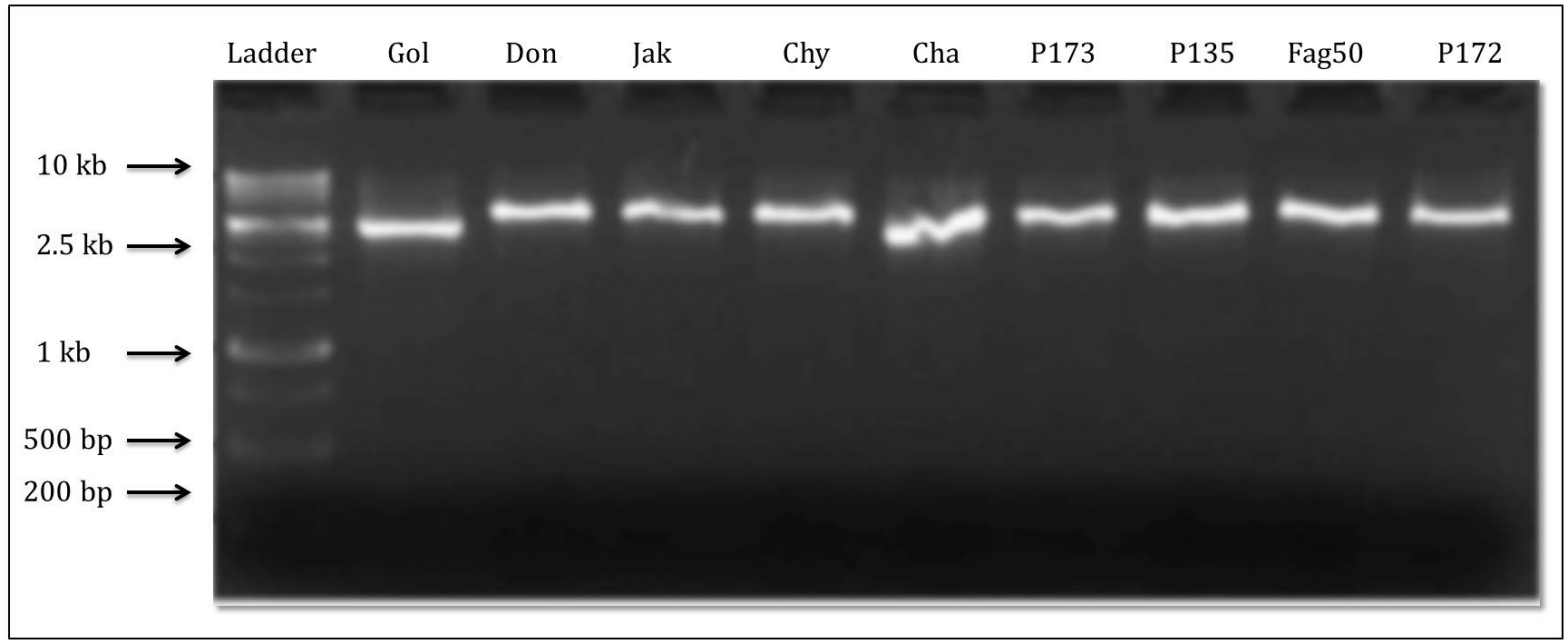
Amplification of PAL gene fragment (approximately 2.6 kb size) from *F*. *tataricum* genotypes (Gol-Golden, Don-Donan, Jak-Jakar, Chy-Chumey, Cha- N7605Chumoa, PI73-PI481673, PI35-PI427235, FAG50-FAG50, PI72-PI481672).

The molecular profiling of PAL gene from different accessions of *F*. *tataricum*, *F*. *esculentum* and *F*. *dibotrys*, led to decipher the species specific allelic sequence variations in the form of SNPs and/or Indels (Fig 2 and S1 Fig).

**Fig 3:**
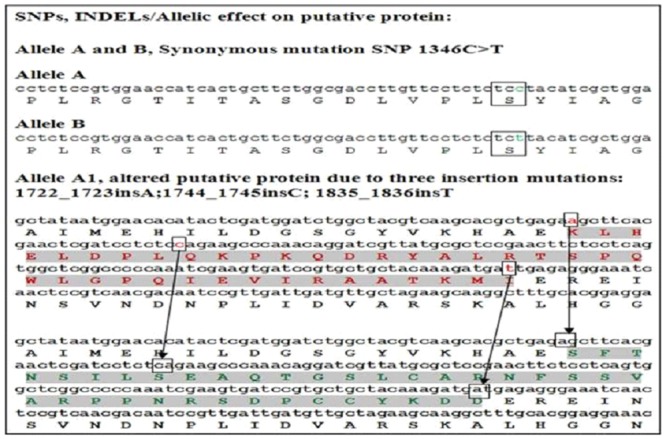
A synonymous mutation and insertion induced frame shift mutation in PAL gene of *F*. *tataricum*.

In addition to the species specific sequence signatures intra-specific variations were also found. SNPs alleles were designated with letters ‘A’ and ‘B’ (Fig 3). Furthermore, two accessions exhibited the presence of three insertions at exon2 (Fig 3 and S2 Fig) causing the variation of a stretch of amino acids with respect to its putative protein and designated as allele ‘A1’ (Table 1).

**Table 1:** *Fagopyrum spp*. PAL gene allele designation with Genbank accession numbers.

*F*. *tataricum* accessions	Allele	Genbank	Size	SNPs/Allele description
*F*. *tataricum* Golden	A	KF286895	860bp	g.[949G>C;1114_1115insG;1346C>T]
*F*. *tataricum* Donan	A	KF286896	811bp	g.[949G>C;1114_1115insG;1346C>T]
*F*.*tataricum* Jakar	B	KF286897	751bp	g.[949C>G;1114_1115insG;1346T>C]
*F*. *tataricum* Chumey	B	KF286898	880bp	g.[949C>G;1114_1115insG;1346T>C]
*F*. *tataricum* Chumoa	B	KF286899	780bp	g.[949C>G;1114_1115insG;1346T>C]
*F*.*tataricum* PI481673	B	KF286900	795bp	g.[949C>G;1114_1115insG;1346T>C]
*F*. *tataricum* PI427235	A	KF286901	720bp	g.[1114_1115insG;1346C>T]
*F*. *tataricum* FAG50	A1	KF680943	2188bp	g.[949G>C;1017A>G;1114_1115insG;1346C>T;1722_1723insA;1744_1745insC;1835_1836insT]
*F*.*tataricum* PI481672	A1	KF408290	2156bp	g.[949G>C;1017A>G;1114_1115insG;1346C>T;1722_1723insA;1744_1745insC; 1835_1836insT]
*F*. *esculentum* Botan	E1	KC792588	620bp	g.[685G>A;697G>C; 707G>A;709C>T;841G>C]
*F*. *esculentum* Karmen	E2	KC792587	983bp	g.[685A>G;697G>C;707G>A;709C>T;841G>C]
*F*. *esculentum* Koto	E1	KC792586	949bp	g.[685G>A;697G>C; 707G>A;709C>T;841G>C]
*F*. *esculentum* Luba	E2	KC792585	983bp	g.[685A>G;697G>C;707G>A;709C>T;841G>C]
*F*. *esculentum* Koban	E2	KC792584	969bp	g.[685A>G;697G>C;707G>A;709C>T;841G>C]
*F*. *esculentum* Svityazyanka	E2	KC792583	983bp	g.[685A>G;697G>C;707G>A;709C>T;841G>C]
*F*. *dibotrys* FCA1_FAG135	F1	KF408292	2141bp	g.[1122C>T;1254G>C;1458C>G;2220C>A; 2432G>C]
*F*. *dibotrys* FCA3_FAG135	F1	KF408293	2133bp	g.[1122C>T;1254G>C;1458C>G;2220C>A;2432G>C]
*F*. *dibotrys* FDD1_FAG142	F2	KF408291	2164bp	g.[1122C>T;1254C>G;1458C>G;2220C>A;2432G>C]
*F*. *dibotrys* FCB3_FAG135	F3	KF680944	2120bp	g.[1122C>T;1254C>G;1458C>G;2220C>A;2432C>G]

### Effect of SNPs/Indel on putative protein

**Fig 4:**
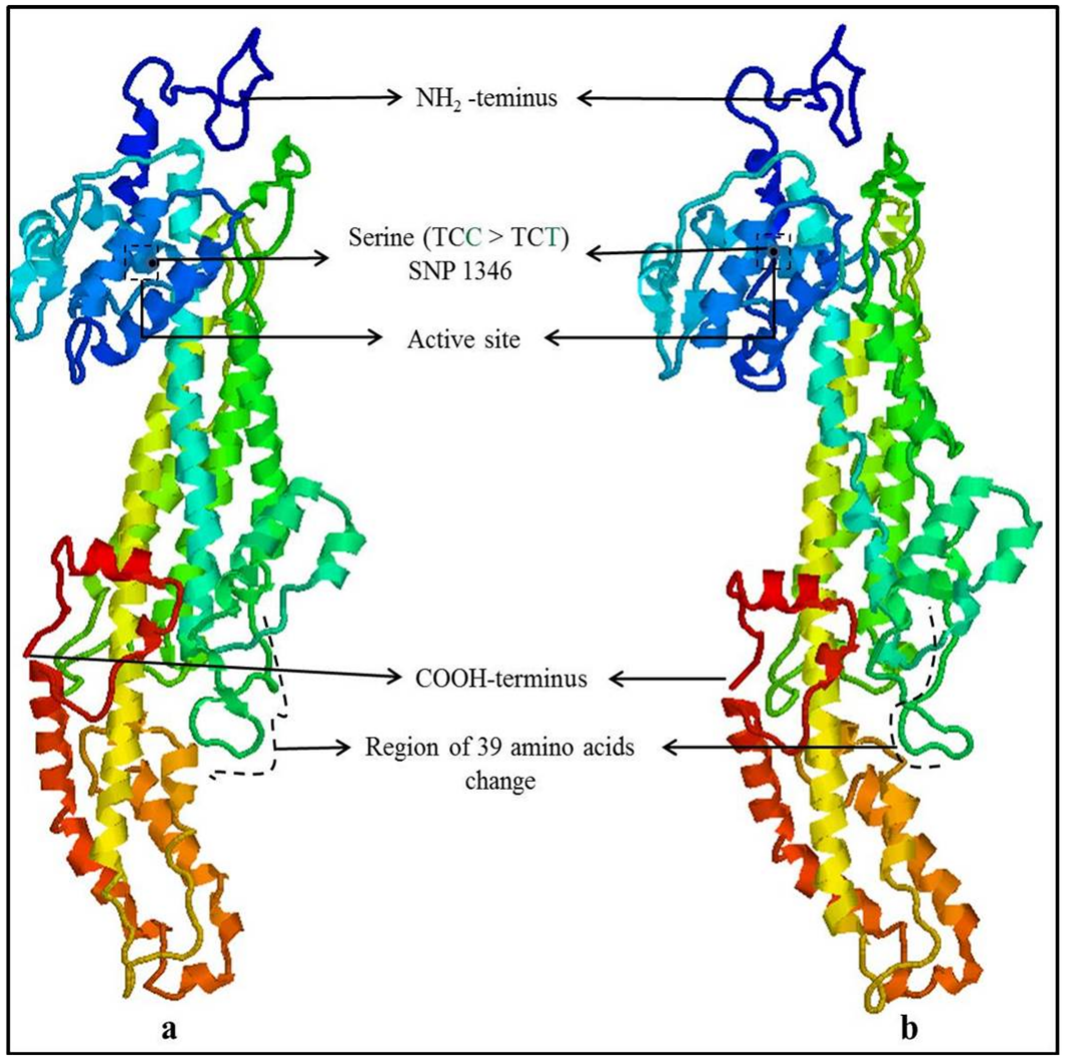
Protein modelling of PAL gene Allele ‘A1’ and PAL gene reference protein describing the structural similarity in active site and other region.

Annotation of Allele ‘A1’ from *F*. *tataricum* sequences revealed three insertion mutations, found to cause frame shift of 39 amino acids in ORF of PAL gene. This frame shift resulted in altered amino acids stretch on putative protein corresponding to exon2 in two *F*. *tataricum* accessions (Figs 3 and 4). Amplified sequences of the other two *Fagopyrum* species were submitted to Genbank and allele designated with the SNPs causing the change of amino acid as shown in Table 1.

### SNP analysis in natural populations of *F*. *tataricum*

**Fig 5:**
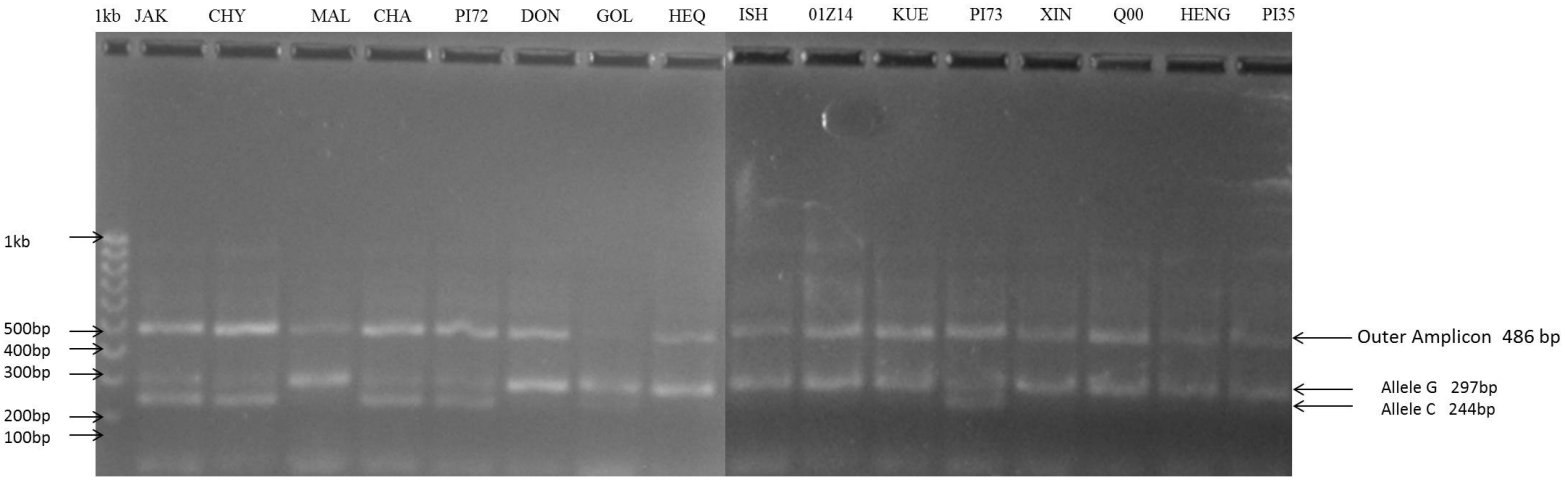
Agarose gel picture depicting the allelic variation among sixteen *F*. *tataricum* varieties differing G>C. Heterozygous (GC alleles with both 297and 244 bp amplicons) and homozygous (G with only 297 bp amplicon). The size of the common outer amplicon was 486 bp. Varieties used: J-Jakar, Y-Chumey, M- C8816 Malong, PI-PI481672, D-Donan, G-Golden, H-Hei Qiao-4, I-Ishisoba, O-01Z100014, K-C9717 Kuer, P3-PI481673, X-Xinong 9909, Q—Q000120, H-Hei Feng, P5-PI427235.

A SNP at 949^th^ base pair position in PAL gene (Fig 5) was found to be present in homozygous and heterozygous conditions in 10 and 6 accessions of *F*. *tataricum* respectively.

**Fig 6:**
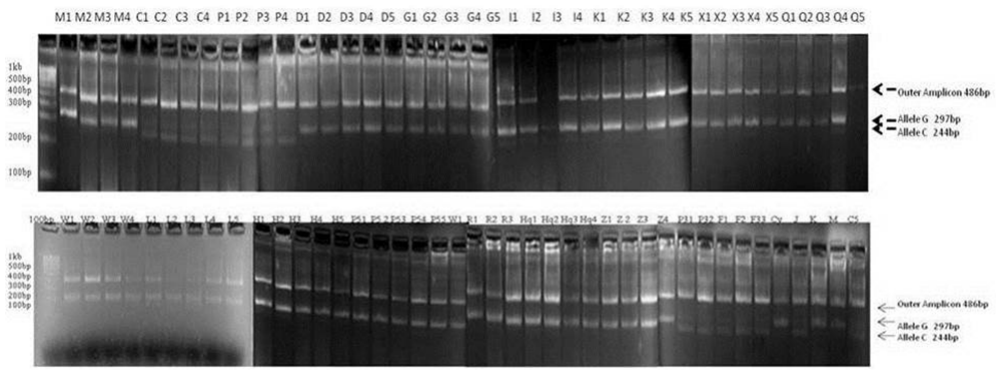
Gel picture differentiating between homozygous and heterozygous alleles at the 949^th^ bp position in *F*. *tataricum* at intra- varietal level. Accessions utilized: M1-M4: C8816Malong, C1-C4: N7605Chumoa, P1-P4: PI481672, D1-D5: Donan, G1-G5: Golden, I1-I4-: Ishisoba, K1-K5: C9717 Kuer, X1-X5: Xinong 9909, Q1-Q5: Q0001120, W1-W4: Wei 93–8, L1-L5: N8614 Lukla, H1-H5:Hei Feng, P51-P55: PI427235, R1-R3: RCAT061058, Hq1-Hq4: Hei Qiao-4, Z1-Z4: 01Z100014, P31-P32: PI481673, F1-F2: FAG 50, F33: FAG 33, Cy: Chumey, J: Jakar, K: Kuer, M: Malong, C5: Chumoa.

Subsequent intra-varietal analysis revealed that among 77 genotypes, 50 and 27 samples showed homozygosity and heterozygosity for this SNP respectively (Fig 6). Further, analysis suggested the existence of strong linkage disequilibrium between SNP positions at 949^th^ and 1346^th^ bp. The SNP at 949^th^ bp had more homozygotes in 16 *F*. *tataricum* varieties of diverse origin as indicated in Fig 6.

### Interspecies sequence analysis

Interspecies polymorphic site analysis revealed the presence of more polymorphic sites in *F*. *dibotrys* followed by *F*. *esculentum* and *F*. *tataricum*. Phylogenetic analysis indicated towards taxonomic closeness of *F*. *tataricum* and *F*. *esculentum*, which was further proved with the presence of relevant SNPs and indel mutations (S3 Fig). However, parsimony informative sites (PIS) with Linkage Disequilibrium (LD) were not collinear to their exact nucleotide positions among these three species, although some PIS were sharing identity with other species (Table 2).

**Table 2:** Parsimony Informative Sites (PIS) and other sites among three species. Legend: PS: Parsimony Informative Sites (PIS), MS: Monomorphic Sites (MS), SV: Singleton Variable, Sites, SV2V: Singleton Variable Sites with Two Variants (SV2V), PIS2V: Parsimony Informative Sites with Two Variants, SV3V: Singleton Variable with Two Variants, PIS3V: Parsimony Informative sites with Three Variants.

Species	Sequences/Sites analysed	PS	MS	SV	PIS	SV2V	PIS2V	SV3V	PIS3V	LD sites	χ^2^
*F*. *tataricum*	8/742bp	3	690	3	2	1	2	0	0	3	1
*F*. *esculentum*	5/742bp	11	723	9	2	9	2	0	0	55	17
*F*. *dibotrys*	5/742bp	19	659	14	5	13	5	1	0	153	18
All aligned	18/742bp	116	553	12	104	12	98	0	6	-	-

**Fig 7:**
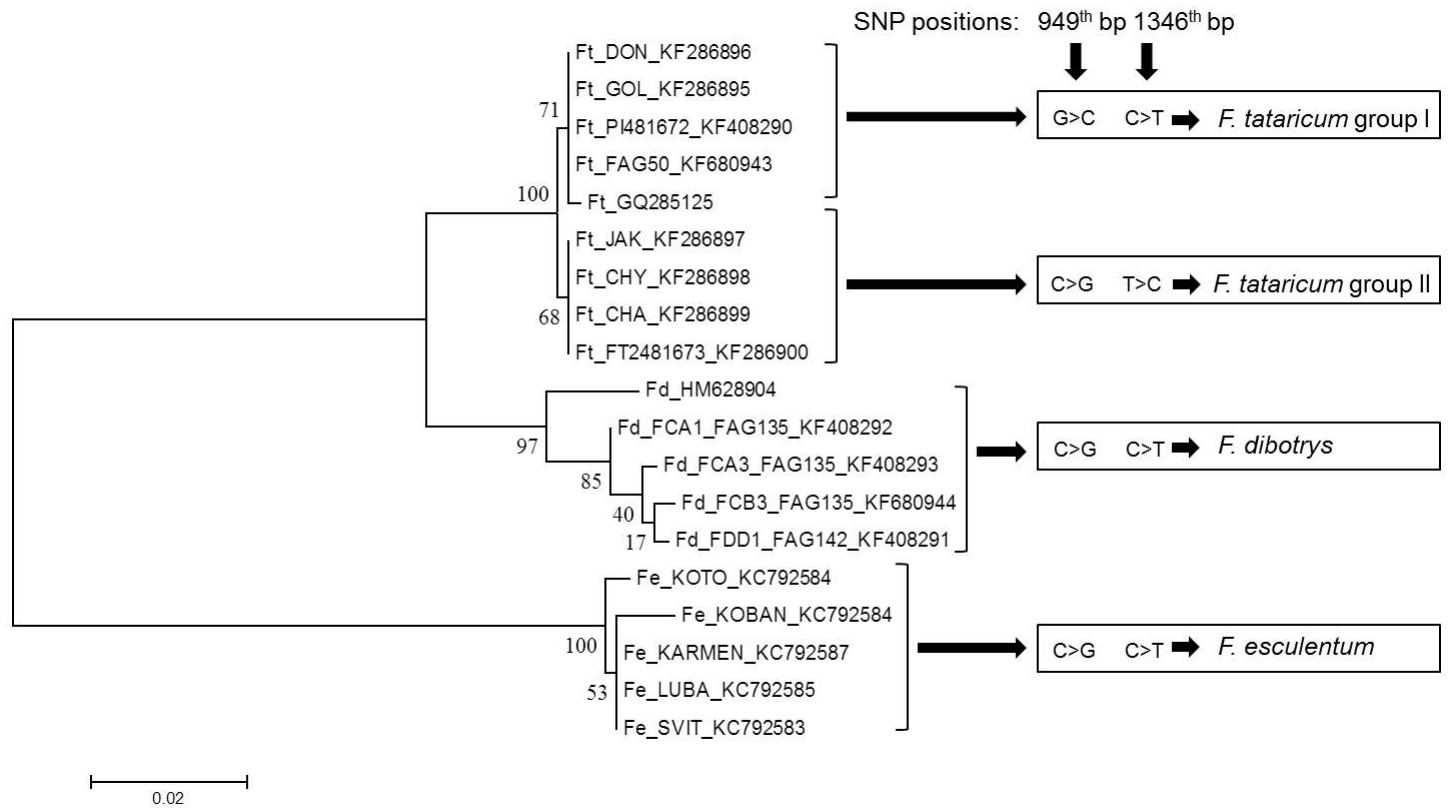
Phylogenetic tree with SNP consistency of each species and accessions. Figure shows that the SNPs in LD with PIS sharing identity among *F*. *tataricum*, *F*. *esculentum* and *F*. *dibotrys*.

**Fig 8:**
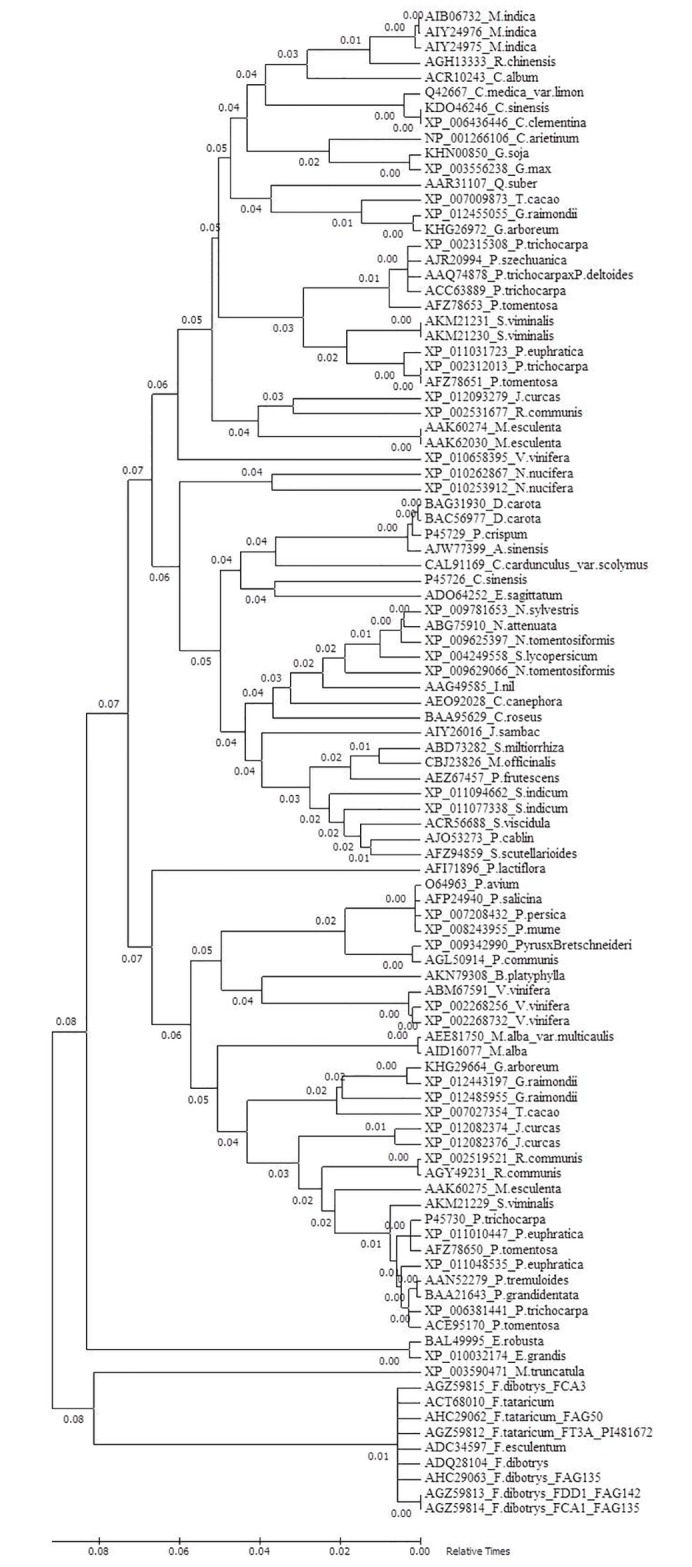
Time tree describing the relative divergence time between *Fagopyrum spp*. and other dicot species.

Gene flow and genetic differentiation resulted into three haplotypes among the accessions of *F*. *tataricum*. Observed homozygous and heterozygous individuals from Tetra primer ARMS PCR were subjected to Hardy Weinberg Equilibrium analysis, which revealed 60 and 33 genotypes with alleles GG and GC with allele frequencies of 62.5% and 37.5% respectively (Fig 5). Further screening of this SNP in intra-varietal genotypes revealed homozygous alleles (64.93%) compared to heterozygous (35.06%) as shown in Fig 6. Phylogenetic study indicated the presence of two *F*. *tataricum* groups sharing each SNP sites (949 and 1346^th^) in LD and PIS separately with *F*. *dibotrys* and *F*. *esculentum* (Fig 7). Divergence time tree clearly explained the relative and early divergence of the ancestor species belonging to the clade of *Fagopyrum spp*. and *Medicago trancatula* than the ancestor species of rest of the dicots clade (Fig 8).

Through sequencing, the heterozygosity at 949^th^ bp position was not identified. However, using Tetra primer ARMS PCR, the presence of heterozygous genotypes (with GC allele) from the natural populations was observed. Interestingly, one of the homozygote allele CC was not found through this method. These results are in agreement with Hardy Weinberg Equilibrium. Practically it was not possible to assess the homozygous individuals with CC allele. This allele was predicted to be in frequency of 0.03 (q^2^ = 0.03) through Hardy Weinberg Equilibrium. The χ^2^ value was 4.33 with significant P-value of 0.0374 (P<0.05). Using the allele frequency of p allele and q allele (0.82 and 0.18), the genotype frequency was calculated according to the Hardy Weinberg Equilibrium (p^2^+2pq+q^2^). Thus, p^2^ = 0.6732, 2pq = 0.1476 and q^2^ = 0.03.

## Discussion

Sequence characterization of the PAL gene was carried out from *Fagopyrum spp*. in this study, which plays an important role in rutin and quercetin bio-synthesis pathway. Species specific sequence signatures were observed showing evolutionary significance of *Fagopyrum* genus as well as putative protein structure. Three insertion mutations and three SNPs were identified in *F*. *tataricum*. Among three SNPs, one was singleton variant and other two are PIS, one at 949^th^ and other at 1346^th^ bp positions. SNPs at 949^th^ and 1346^th^ bp position were in intron1 and exon 2 respectively in the PAL gene.

The three insertion mutations in PAL gene caused a variation of stretch of 39 amino acids in exon2 of ORF in comparison with reference PAL protein, ACT68010 (Figs 3 and 4). These insertion mutations caused frame shift of 39 amino acids resulted into different protein isoform as implicated in this present study in accordance with the previous reports [21, 22]. Altered protein due to change of 39 amino acids likely resulting for the evolution of adaptive proteins [23] and may cause structural and functional changes. Theoretical predictions of physico-chemical properties revealed that the protein of allele ‘A1’ (altered protein of 39 amino acids) possessed 57 positively charged residues (Arginine + Lysine) with 6.19 theoretical isoelectric focusing point (pI), while the reference protein possessed 53 positively charged residues with 5.81 pI. The instability index of the variant region with 39 amino acids of allele ‘A1’ alone considered as unstable one, as instability index (II) calculated was 76.08, which exceeded the instability index limit of 40 [24]. The transition but synonymous mutation observed at 1346^th^ position did not change the amino acid ‘serine’. Further, comparison of the putative protein of PAL allele A1 (AHC29062) in reference to PAL putative protein (Protein ID: ACT68010) and indicated for no change in active site (GTITASGDLVPLSYIAG). However, protein modelling suggested a significant alteration in the protein structure and thereby the possible alteration of physico-chemical properties.

**Fig 9:**
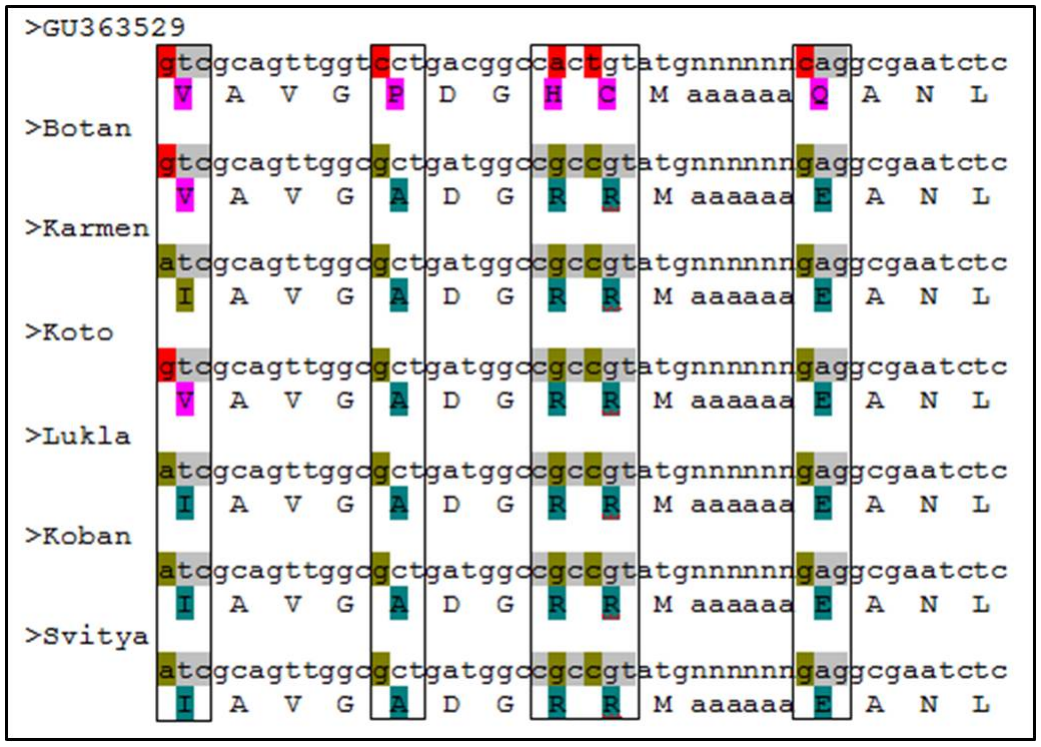
Description of conservative and non-conservative amino acid change due to SNP mutation in PAL gene of *F*. *esculentum*.

**Fig 10:**
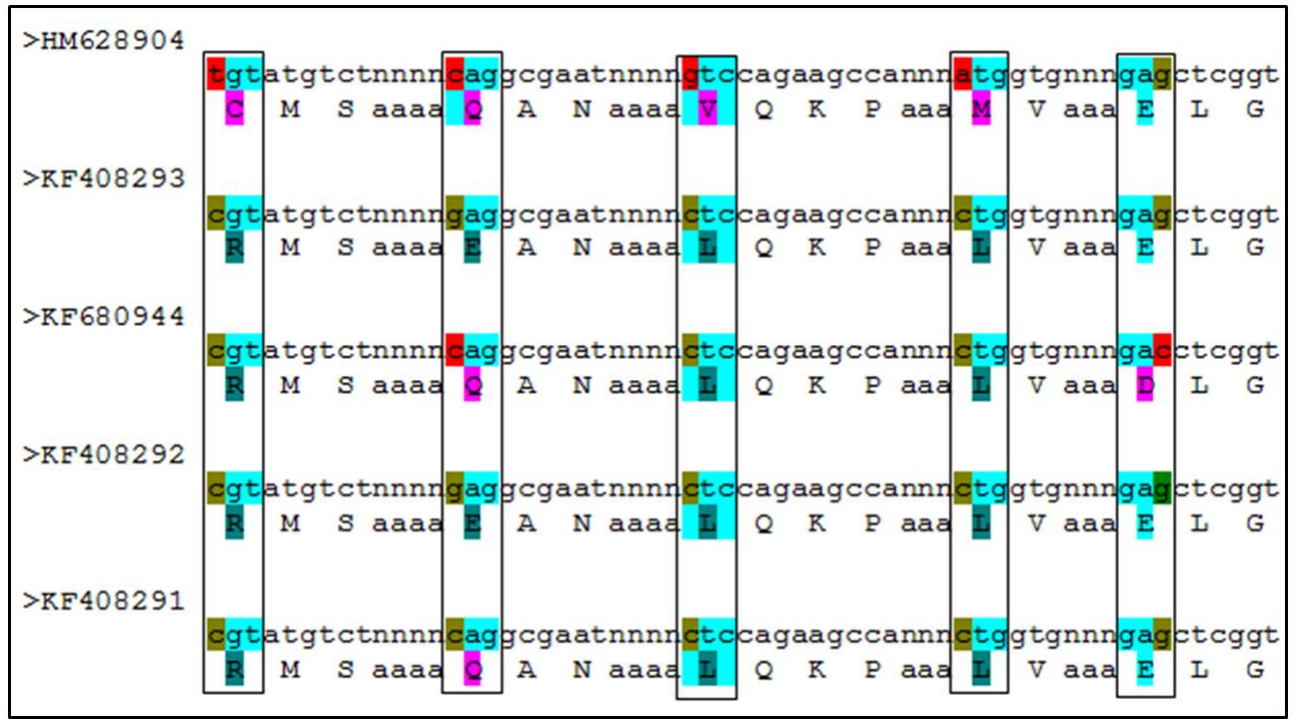
Description of conservative and non-conservative amino acid change due to SNP mutation in PAL gene of *F*. *dibotrys*.

The amino acid change in exon2 of *F*. *esculentum* is shown in Fig 9. There were five amino acids change and two of them were conservatively altered (Glutamine to Glutamic acid, Valine to Isoleucine) and changes in other three amino acids were non conservative (Proline to Asparagine, Histidine to Arginine, Cysteine to Arginine). Similarly, five amino acid changes were observed in *F*. *dibotrys* (Fig 10). In *F*. *dibotrys* exon2, the SNPs caused two conservative changes in amino acids (Glutamine to Glutamic acid, Glutamic acid to Aspartic acid), whereas other SNPs caused non conservative alteration (Cysteine to Arginine, Valine to Lysine, Methionine to Lysine). Although, the positions of amino acid change were not collinear between *F*. *esculentum* and *F*. *dibotrys*. In *F*. *tataricum*, no amino acid change was detected from the observed synonymous mutation/SNPs variation, while three insertion mutations caused the change of long stretch amino acids. Apart from these non-silent mutations, there were more than 30 SNPs silent mutations observed in both *F*. *esculentum* and *F*. *dibotrys*, while in *F*. *tataricum* only one silent mutation was observed. Overall, we found more SNP mutations in allogamous species *F*. *esculentum* and *F*. *dibotrys*, than autogamous *F*. *tataricum*. Conversely, indel mutations were observed only in *F*. *tataricum* (not in *F*. *dibotrys* and *F*.*esculentum*) which caused a major change in putative protein (Fig 3). The SNP and indel mutations observed in different *F*. *tataricum*, *F*. *dibotrys* and *F*. *esculentum* indicate towards the evolutionary role of PAL gene in *Fagopyrum* spp.

The sequences of *F*. *tataricum* were represented as two sub-groups (group 1 and 2) according to the 949^th^ and 1346^th^ bp SNPs (Table 3). Genetic diversity within and between the two groups revealed that the group one is more diverged as compared to sub-group two. Genetic differentiation of both assigned sub-groups was statistically significant with pairwise comparison. Haplotype based statistics for the genetic differentiation of these two groups was significant with PM test (Table 3). This finding was further supported by Fst estimate and effective migrants (Nm) indicated towards an absolute migration with low gene flow (Table 3). Similar trend of haplotype diversity was also previously reported [25]. These results clearly indicated the phylogenetic importance of two tightly linked PIS at 949^th^ and 1346^th^ bp SNP positions.

**Table 3:** Genetic diversity, differentiation and gene flow analysis in *F*. *tataricum*.

		Genetic Diversity				
Sub-groups	Sequences	Segregating sites	Haplotypes	Hd	K	PiJC
SNP1 G>C and C>T	5	1	2	0.4	0.4	0.0006
SNP2 C>G and T>C	4	-	1	-	-	-
Both SNPs	9	3	3	0.66	1.33	0.0019
		Genetic differentiation of both SNP Sub-groups				
	χ^2^	Hst	Kst	Kst*	Z*	Snn
Estimated value	9	0.64	0.83333	0.76873*	2.0644	1*
Value of PM test	0.0111*	0.0190*	0.0100 *	0.0100 *	0.0100 *	0.0100 *
		Gene flow of both SNP Sub-groups				
	Haplotype data		Sequence Data			
	Gst		DeltaSt	GammaSt	Nst	Fst
Estimated value	0.64		0.00145	0.85	0.9092	0.9091
Nm	0.14		0.04	0.04	0.02	0.03

Hd-Haplotype Diversity, K- Average Nucleotide Differences, PiJC- Nucleotide diversity with Jukes Cantor corrections, χ^2^- Chi Square test, Hst- Haplotype based statistics, Kst- Sequence based statistics, Z*- Rank Statistics, Snn- Near Neighbour Statistics, Gst- Differentiation of population, GammaSt- Gamma Statistics, Nst- N statistics, Fst- Fixation index, Nm- Effective number of migrants, PM- Permutation test with 1000 replicates,

* 0.05<P.

Putative PAL gene protein of *Fagopyrum spp*. (generated in our study) was aligned with the protein of PAL gene from other dicot spp. PAL protein, which aligned from *Fagopyrum* spp. and other dicots led to identify the conserved signature motif ‘GTITASGDLVPLSYIAG’. Further, we calculated relative divergence time (0.8), which revealed an early divergence of the ancestor species of the clade of *Fagopyrum* spp. and *Medicago trancatula* from the ancestor species of the clade other dicot spp. (0.7) subjected to analysis. Besides, within a clade, the divergence time revealed an early divergence of *Fagopyrum* spp. (0.1) than *Medicago trancatula* (0.0). It is noteworthy fact that both *M*. *trancatula* and *Fagopyrum* spp. are well known for rutin production [26, 6], whereas in most other dicots, it has been predominantly associated with lignin and anthocyanin production [27, 28, 29]. In particular, 8 amino acids were identical between these two species corresponding to *F*. *tataricum* PAL protein 642^th^ to 652^th^ amino acid positions: ‘ARTLYNNGASG’ rather than other species. Therefore, protein sequence alignment clearly revealed the close proximity of amino acids of *Fagopyrum spp*. with *Medicago trancatula*, which is highly likely associated with rutin bio-synthesis pathway.

There were two SNPs in *F*. *tataricum* (SNP at 949^th^ and 1346^th^ bp position) showing LD and PIS and one of them (SNP at 949^th^ bp position) showed association with agronomically important traits. SNPs at 949^th^ and 1346^th^ bp were located in intron (only intron of this gene) and exon2 respectively. The SNP at 949^th^ position was always found to be in LD with 1346^th^, a mutation in the first site is always paired with the presence of SNP in the second site (i.e. 1346^th^ bp). Interestingly, heterozygosity at these sites (SNP at 949^th^ and 1346^th^ bp position) showed correlation with increased seed number, reduced plant height and 100-kernel weight (Table 4). It is a well-established fact that SNPs at splicing sites or branch points of intron may affect the splicing of intron and exon. As the result mRNA transcript may be abnormal, because of these kinds of mutational consequences of important sites at intron. But in this study, we found a mutation apart from these splicing sites or branch points, so functionally it has no direct role, while the SNP/mutation in intron (949^th^ position) always paired with 1346^th^ exon2 SNP due to LD. If there is alteration in SNP at 949^th^ bp (intron) then there will be alteration in exon at 1346^th^ bp due to LD. Based on these facts we hypothesize that that SNP mutation in exon have ‘functional agronomic’ role. However a definitive test would further confirm this.

**Table 4:** *F*. *tataricum* intraspecies accessions zygosity and phenotypic traits assessment.

Traits	AVS (numbers)	100 KW (mg)	APH (cm)
HZ—GG	256.44	2536.94	205
HT—GC	282	2056.6	151.6
Std. Dev	4.38	4.43	4.05
Sum Sq	3.938	3.937	3.437
Mean Sq	0.28	0.28	0.28
LSD mean	1.43	1.43	1.43
LSD CV	3.50e-15	7.36e-15	28.39
MSerror	2.54e-33	1.12e-32	0.16
F test	1.109e+32 ***	2.516e+31***	1.719 Ns

AVS- Average Seeds Per Plant, 100KW- 100 Kernel Weight, APH—Average Plant Height;

***0.001<P, Ns-Not Significant.

Numerous studies have been focused on SNP analysis of PAL gene in different plant species to improve the yield with reference to rutin, anthocyanin, lignin or relevant metabolites [30, 31, 32, 33]. Among *Fagopyrum* spp. total flavonoid content is commonly higher in *F*. *tataricum* than *F*. *esculentum*. Among released *F*. *tataricum* varieties, ‘Donan’ is very popular and known for high thousand kernel weight as revealed in our study (data not presented). This variety can be utilized as a potential germplasm source for medicinal application.

### Polymorphic sites in *Fagopyrum* spp. at inter and intra species level

Through interspecies sequence analysis of the three *Fagopyrum* species, we identified PIS and other useful sites (Table 2). Disparity index revealed the existence of homogenous substitution pattern between *F*. *tataricum* and *F*. *dibotrys* with significant heterogeneity between *F*. *esculentum* and *F*. *dibotrys* (S1 Table). Distance matrix index values also revealed that the distance between *F*. *tataricum* and *F*. *esculentum* is more than *F*. *dibotrys*. The distance index between *F*. *dibotrys* and *F*. *tataricum* was between 4–5%, while the distance index with between *F*. *dibotrys* and *F*. *esculentum* was 15–16% (S2 Table, Fig 7). Similar results were presented in previous reports [34].

*F*. *esculentum* and *F*. *tataricum* had two PIS, while in *F*. *dibotrys* four PIS were observed. Intra-specific SNPs were maximum in *F*. *dibotrys* (18) followed by *F*. *esculentum* (11) and lest in *F*. *tataricum* (3). SVs were also least in *F*. *tataricum* than other two allogamous species. In these three species balancing selection maintained the monomorphic sites at 553 positions and thus the variations of only 116 positions allowed to discriminate these species. In contrast, the adaptive mutation reduced the variations of these 553 positions, which are remaining unchanged during evolution (Table 2). Besides, the gene exhibited significant variation with 42 bp deletion in *F*. *tataricum* and *F*. *dibotrys* as shown in S3 Fig (corresponding to the insertion in *F*. *esculentum*).

In *F*. *tataricum* three pair of sites with LD was observed and among them, the one between 949^th^ and 1346^th^ bp was statistically significant (S3 Table). Allelic pattern at this LD site in PAL gene have been depicted in Fig 7. LD event in *F*. *tataricum* classified this species in two different groups (groups I and II). The SNP allele of *F*. *tataricum* group II at 949^th^ bp (Cross species comparison site 952) showed identity in *F*. *esculentum* and *F*. *dibotrys* at this locus, indicating that this allele was contributed to *F*. *tataricum* by *F*. *dibotryis*/*F*. *esculentum*, while group I allele from some other progenitor. Similar observation for *F*. *tataricum* group I allele at 1346^th^ bp (cross species comparison site 1395) supported to above mentioned conclusion. Noticeably, these PIS and/or recombinations were found within 400 bp region of PAL gene. There were other LD events present in this gene among different *Fagopyrum* spp. as indicated in the S4 Fig. LD sites were more in allogamous species (*F*. *dibotrys* and *F*. *esculentum*) than *F*. *tataricum*. *F*. *tataricum* group II was closer to *F*. *dibotrys* than group I as shown in Fig 7. It clearly revealed the importance of SNPs with LD and PIS of PAL gene in evolution. These SNP and indel variations clearly indicated that *F*. *tataricum* is more closely related to *F*. *dibotrys* than *F*. *esculentum* (Fig 7 and S3 Fig). The species specific sequence signature in PAL gene of three *Fagopyrum* spp. has emphasized the phylogenetic importance of this gene.

There were three types of inter-specific SNPs: (i) which represented LD and PIS (ii) other which showed only LD and not PIS and (iii) those which only represented PIS. With reference to the SNPs, which represented both LD and PIS in *F*. *tataricum* were species specific i.e. across the species they were not comparable (Table 2). SNPs in two positions, which showed LD of *F*. *tataricum*, are not sharing identity in other species, while one SNP among these two were sharing identity in either species. SNPs with PIS alone shared more identity between *F*. *tataricum* and *F*. *dibotrys* than *F*. *esculentum*. These results indicate that species specific SNPs are under selection pressure, when they are in LD. The breakage of LD due to mutation, genetic drift and absence of selection pressure might disturb these SNPs. SNP at the 949^th^ bp position had two alleles ‘GG’ and ‘CC’. Interestingly, in the natural population of *F*. *tataricum* we could detect only one homozygote ‘GG’ and heterozygote ‘GC’. The ‘CC’ homozygote was neither identified through sequencing the gene nor through following Tetra primer ARMS PCR strategy. Following the Hardy Weinberg Equilibrium, we predicted the frequency of ‘CC’ homozygote (0.03%) to be rare. This was the most probable reason for not identifying the rare allele ‘CC’ in present study.

Present study provides an in-depth sequence characterization of PAL gene in *Fagopyrum* spp. which is known for its medicinal value. The sequence information concerning the SNPs/alleles can be used for the identification of elite cultivars from germplasm collections of *F*. *tataricum* and related species within the genus Fagopyrum as well as the species from other genus of plant kingdom. Certain insertion/deletions caused major variations of amino acids in *F*. *tataricum* possibly due to genomic plasticity events in this species, which harbored beyond normal mutations and thus caused enormous variations. Comparative genomics of these kinds of alleles with other species will excavate the rare mutations in other species. Overall analysis clearly suggested towards an evolutionary significance of PAL gene in the genus *Fagopyrum*. Informations presented in this report can be efficiently utilized in genetic improvement of *Fagopyrum* spp. with respect to its medicinal relevance.

## Materials and Method

### Genotypes and DNA extraction

Sixteen accessions of *F*. *tataricum* were utilized for the screening of inter and intra-specific diversity. To facilitate the understanding of the evolutionary relationship, five accessions of *F*. *esculentum* and five of *F*. *dibotrys* were also included. The genetic material was either obtained from different sources as shown in Table 5.

**Table 5:** Buckwheat varieties and accessions utilized for the screening of inter- and intra-specific diversity: origin and seed source.

Species/variety	Origin	Source
***Fagopyrum tataricum***		
Golden	Bosnia-Hercegovina	Parco Scientifico e Tecnologico del Molise, Campobasso, Italy
Q0001120	China	Department of Biology, Honghe University, Yunnan, China
PI481672, PI481673	Bhutan	Northeast Regional PI Station, USDA, Agricultural Research Service, Plant Genetic Resources Unit, Geneva, New York, USA
PI427235	Nepal	Northeast Regional PI Station, USDA, Agricultural Research Service, Plant Genetic Resources Unit, Geneva, New York, USA
Hei Feng, Hei Qiao-4, Wei 93–8, Xinong 9909	China	Hodowli Róslin Palikije, Wojciechów, Poland
01Z5100014	USA	Department of Gene Bank, Division of Genetics and Plant Breeding, Research Institute of Crop Production, Prague-Ruzyne, Czech Republic
C8816Malong, C9717 Kuer	China	Plant Germ-Plasm Institute, Graduate School of Agriculture, Kyoto University, Japan
N7605Chumoa, N8614 Lukla	Nepal	Plant Germ-Plasm Institute, Graduate School of Agriculture, Kyoto University, Japan
Donan, Ishisoba	Japan	Plant Genetic Resources Laboratory, Dept. of Upland Agriculture, National Agricultural Research Center for Hokkaido Region, Shinsei, Memuro-cho, Kasai-gun, Hokkaido, Japan
FAG 50	China	The Leibniz Institute of Plant Genetics and Crop Plant Research (IPK) in Gatersleben, Germany
RCAT 061058	Unknown	The Institute for Agrobotany at Tápiószele, Hungary
***Fagopyrum esculentum***		
Karmen, Svityazyanka	Belarus	RUP The Institute of Arable Farming and Plant Breeding of the National Academy of Sciences of Belarus, Zhodino, Minsk, Belarus
Koban, Koto	Canada	Kade Research Ltd., Morden, Manitoba, Canada
Luba	Poland	Hodowli Róslin Palikije, Wojciechów, Poland
***Fagopyrum dibotrys***		
FAG 135	Unknown	The Leibniz Institute of Plant Genetics and Crop Plant Research (IPK) in Gatersleben, Germany
FCA3_FAG135	Unknown	IPK, Gatersleben, Germany
FCB3_FAG135	Unknown	IPK, Gatersleben, Germany
FCA1_FAG135	Unknown	IPK, Gatersleben, Germany
FDD1_FAG142	Unknown	IPK, Gatersleben, Germany

In order to analyse the intra-varietal zygosity, about four genotypes of each *F*. *tataricum* accession were germinated in petri plates, transferred to pots and grown in a greenhouse.

For each genotype, approximately 100 mg of fresh leaves were collected from 4 weeks old plantlets and ground with liquid nitrogen. Total DNA was extracted by CTAB method [35], quantified using MaestroNano Micro-Volume Spectrophotometer (Cat. No. MN-913, Maestrogen) and further diluted with sterile distilled water to obtain a DNA template with a concentration of 50 ng/μl. Similar methodology was followed for the extraction of DNA from individual genotypes of *F*. *esculentum* and *F*. *dibotrys* accessions as mentioned in the Table 5.

### Polymerase Chain Reaction and Sequencing

Specific forward and reverse primers for *F*. *tataricum* PAL gene were designed (S4 Table) using reference sequence available at GenBank [13]. Primers, synthesised by Sigma Aldrich S.r.l. (Milano, Italy), allowed amplifying the whole gene, from start to stop codon, within a single Polymerase Chain Reaction (PCR). Alternatively, additional couples of primers were also designed to anneal with different regions, so that the fragments obtained, when overlapped, would cover the whole length of the gene. The PCR reaction volume was fixed at 25 μl and included the following reagents: 2 μl of dNTP 200 uM, 1.5 μl of 3 mM MgCl_2_, 2.5 μl of 1X Reaction buffer, 0.2 μl of 1 Unit Bioline *Taq*, 1 μl of 1 pM Forward primer, 1 μl of 1 pM Reverse primer, 15.8 μl of sterile distilled water and 1 μl of DNA template.

The PCR amplification was performed on a Mastercycler^®^ pro (Eppendorf) thermocycler using the following cycling program Initial denaturation at 94°C for 5 minutes, 35 cycles consisting of 1 minute denaturation at 94°C, 1 minute annealing at 57°C and, 1.5 minutes extension at 72°C, and final extension at 72°C for 10 minutes. Samples were stored at 4°C overnight and subsequently added with 2 μl of MaestroSafe Nucleic Acid loading dye (Cat. No. MR-031201, Maestrogen). Amplified fragments were resolved using 2% agarose gel electrophoresis at 90 V for 90 minutes. Each time the expected size band was visualized through an UltraSlim LED Illuminator (Cat. No. SLB-01W, Maestrogen) identified thanks to the comparison with a 1 kb molecular-weight size marker (DNA ladder) (AccuRuler) and excised from the gel with the aid of a clean scalpel. Excised fragments were purified using a Sigma Aldrich GenElute agarose gel purification kit following the manufacturer’s directions.

The concentration of purified fragments was measured with a MaestroNano Micro-Volume Spectrophotometer (Cat. No. MN-913, Maestrogen) and diluted to 56 ng/ μl. 1 μl of the solution was added with 13 μl of sterile distilled water and 1 μl of 10 μM appropriate primer. The reaction mixture obtained was sent for sequencing with AB1 sequencer by Ylichron/Genechron, Rome. Previously synthesised internal primers were used for sequencing (S4 Table).

### Utilization of the sequences for SNPs identification and phylogenetic analysis

Chromatograms were screened using Finch TV (Geospiza Inc., USA) chromatogram viewer software. Sequences of the expected fragment were aligned using Clustal W [36] and the presence of SNPs and insertion deletion mutations was manually detected. Among these the potential SNP (949, G>C) with Parsimony Informative Site (PIS) was selected and utilised as a basic platform for designing Tetra primer ARMS PCR. Phylogenetic analysis and Relative Divergence Time were done using MEGA (Molecular Evolutionary Genetic analysis software) [37]. Using PAL gene/alleles generated in this study and with reference gene sequences from NCBI, a Phylogenetic tree was constructed through Maximum Likelihood method with Jukes Cantor (JC) model and 1000 bootstrap resampling. Besides, *F*. *tataricum* putative PAL protein (AHC29062) was subjected to BLASTp against non-redundant (nr) protein database at NCBI and 98–100% query coverage with 85%-99% similarity range based dicot orthologous sequences were retrieved and aligned using Clustal X [36]. Subsequently excluding gaps and missing parameter, Time tree was generated through RelTime using Maximum Likelihood method with Jones-Tailor-Thorns (JTT) model and 1000 bootstrap resampling [38]. Nucleotide substitutions were assessed through disparity index [39] using Monte Carlo test with 500 replicates. Genetic analysis was done using a computational algorithm Gamma statistics for gene flow estimates of haplotypes [40], DeltaST [41], Nst [42], Fst [43] of sequence gene flow estimates and other analysis were done using DNAsP V5 [44]. The Hardy Weinberg Equilibrium was assessed with OEGE, Hardy-Weinberg Equilibrium calculator [45] using number of homozygous and heterozygous genotypes resulted from Tetra primer ARMS PCR. Tetra primers were designed using the tools/program available at the web server http://primer1.soton.ac.uk/primer1.html [14]. Tetra primer ARMS PCR reaction master mix and primers are shown in S5 and S6 Tables respectively. Inter and intraspecific SNPs with PIS were subjected for evolutionary analysis. Tetra primers of the SNP locus 949 are Forward Outer Primer 949 (FOP 949), Reverse Outer Primer 949 (ROP 949), Forward Inner Primer 949G (FIP 949G) and Reverse Inner Primer 949C (RIP 949C). To maximize the allele amplification, a single base pair mismatch was introduced at 3’ of inner primers according to the Tetra primer ARMS PCR designing strategy The amplification of the position 949 was achieved with simple profile PCR program as follows: Step 1: 94°C Initial denaturation for 5 Minutes, Step 2: 35 cycles of 25 seconds of denaturation at 94°C, 35 seconds of annealing at 60°C, 30 seconds of extension at 72°C, Step 3: Final extension of 10 minutes at 72°C. The outer band amplicon size was size 484 bp, the G allele and C allele amplicon size was 297bp and 244 bp respectively. In order to improve the amplification the concentration of outer and inner primers were maintained at 1:2 ratio (10 μM of Outer primer and 20 μM of Inner Primer). The amplified products were resolved and visualized using 5% agarose gel. Further primers were designed and the same methodology was applied to amplify either whole PAL gene or fragments of *F*. *esculentum* and *F*. *dibotrys* and clear chromatogram derived FASTA file fragments were assembled using CAP3 [46].

Protein modelling was done using Geno3D [47] and visualized and annotated with Rasmol [48]. Active site finding was done with Scanprosite tools and the documentation of protein physico- Chemical parameters including instability index was calculated using Protparam tool at Expasy server http://web.expasy.org/tools/protparam/protparam-doc.html [24].

### Phenotypic analysis study

All phenotypic and genotypic data was imported to MS-Excel and the results were compared with homozygous and heterozygous alleles for a SNP position with parsimony informative site and linkage disequilibrium. The statistical analysis of phenotypic traits with respect to zygosity was done using R program [49].

## Conclusion

*F*. *tataricum* and *F*. *esculentum* are medicinally important species besides the nature of being pseudocereal food resource crops. Genetics and genomics studies are being focused widely for these two species to enhance their medicinally important flavonoid compounds rutin and quercetin. We here report that the medicinally important PAL gene has an evolutionary significance in *Fagopyrum* spp. Further, we also provided a detailed sequence characterization of this gene which led to identify novel SNP and indel variations. Informations generated in this report can be efficiently utilized in genetic improvement of the under-utilized domesticated *Fagopyrum* spp. for nutraceutical food resource.

## Supporting Information

S1 Fig Gel picture and depiction of amplified fragments of PAL gene in *Fagopyrum tataricum*. (TIF)

S2 Fig *F*. *tataricum* accessions SNP/Indel observation from corresponding chromatograms, arrows indicating the position of SNP or Indel. Legend: **a)** SNP 949^th^ position G>C **b)** SNP 1346^th^ position C>T **c)** SNP 1017^th^ position G>A **d)** Insertion of G between 1114^th^ and 1115^th^ positions **e)** Insertion A between 1722^nd^ and 1723^th^ **f)** Insertion C between 1744^th^ and 1745^th^ positions **g)** Insertion of T between 1835^th^ and 1836^th^ positions.
(TIF)

S3 Fig Comparable deletions between *Fagopyrum tataricum* and *Fagopyrum dibotrys*. (TIF)

S4 Fig Linkage Disequilibrium sites inside PAL gene in *Fagopyrum tataricum* (A), *Fagopyrum esculentum* (B) and *Fagopyrum dibotrys* (C). (TIF)

S1 Table Disparity Index (I_D_) test, 1. Fd_KF408292, 2. Fd_KF680944, 3. Fd_KF408293, 4. Fd_KF408291, 5. Fd_HM628904, 6. Ft_ GQ285125, 7. Ft_KF680943, 8. Ft_ FTPI481672, 9. Ft_KF286897, 10. Ft_ KF286898, 11. Ft_ KF286899, 12. Ft_KF386900, 13. Ft_KF286896, 14. Ft_KF286895. Bold letters are significant, P<0.05.
(DOCX)

S2 Table Distant Matrix calculated with Kimura-2-Parameter using MEGA. (DOCX)

S3 Table Linkage Disequilibrium with pair of parsimony informative sites. Nucleotides represented in capital letters, are the sites in LD and corresponding positions of other species in represented in small letters. D: Linkage Disequilibrium, D’ = Correlation Coefficient of the pairing SNPs, R^2^ = Coefficient of determination, χ^2^ = Chi square test, B = Bonferroni corrections, F = Fisher test, **P<0.01, *P<0.05.
(DOCX)

S4 Table Primers utilised for PCR amplification and sequencing. (DOCX)

S5 Table PCR master mix concentration with Tetra primers adopted for SNP allele validation. (DOCX)

S6 Table Tetra primers of SNP position 949^th^ bp. (DOCX)
