# Supplementary figures and images for: Genomic Characterization of Phenylalanine Ammonia Lyase Gene in Buckwheat
Source: PLoS One. 2016 Mar 18;11(3):e0151187. doi: 10.1371/journal.pone.0151187 (PMC4798664; doi:10.1371/journal.pone.0151187)

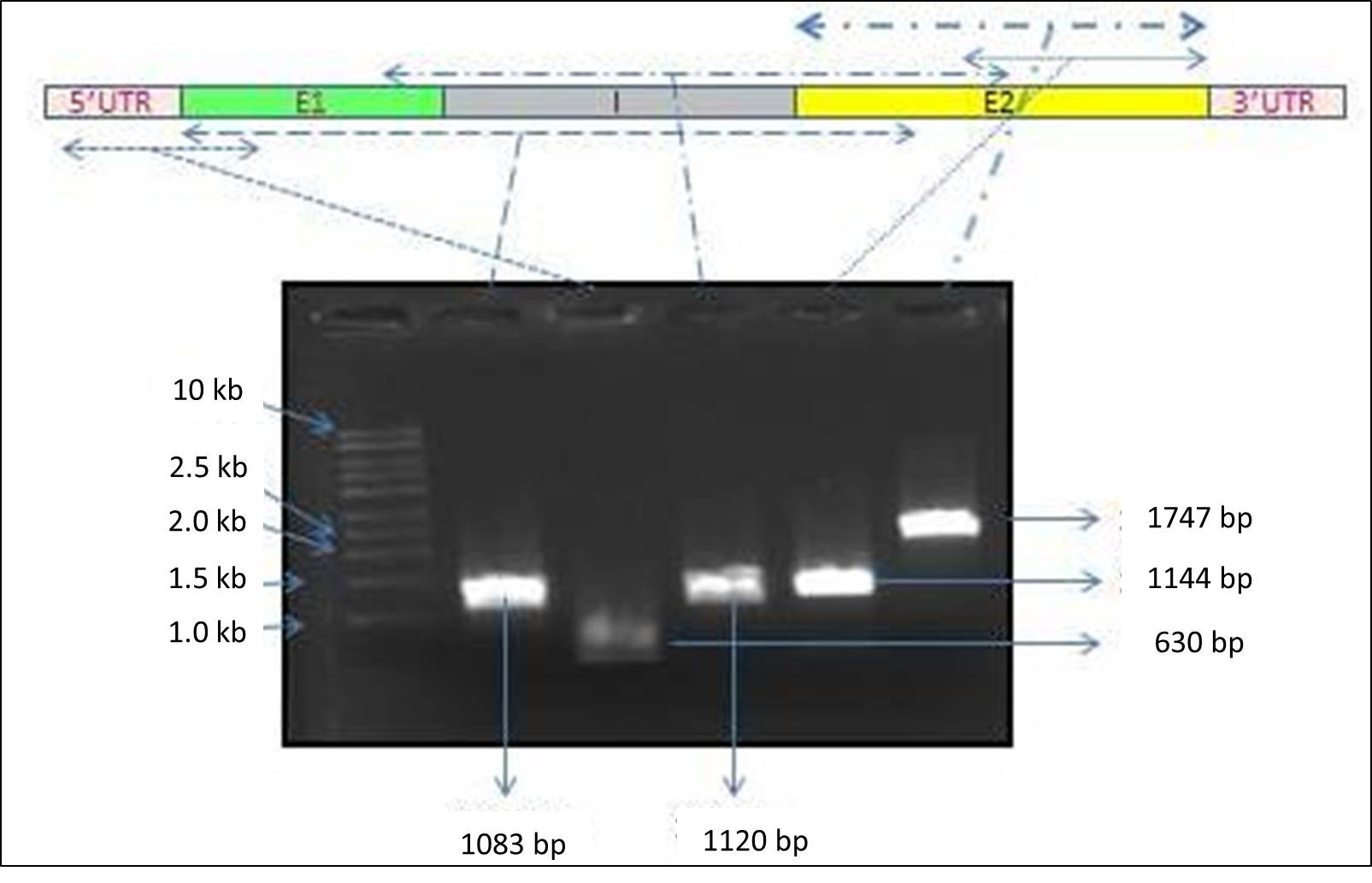

Supplement: S1 Fig — (TIF) [file pone.0151187.s001.tif]

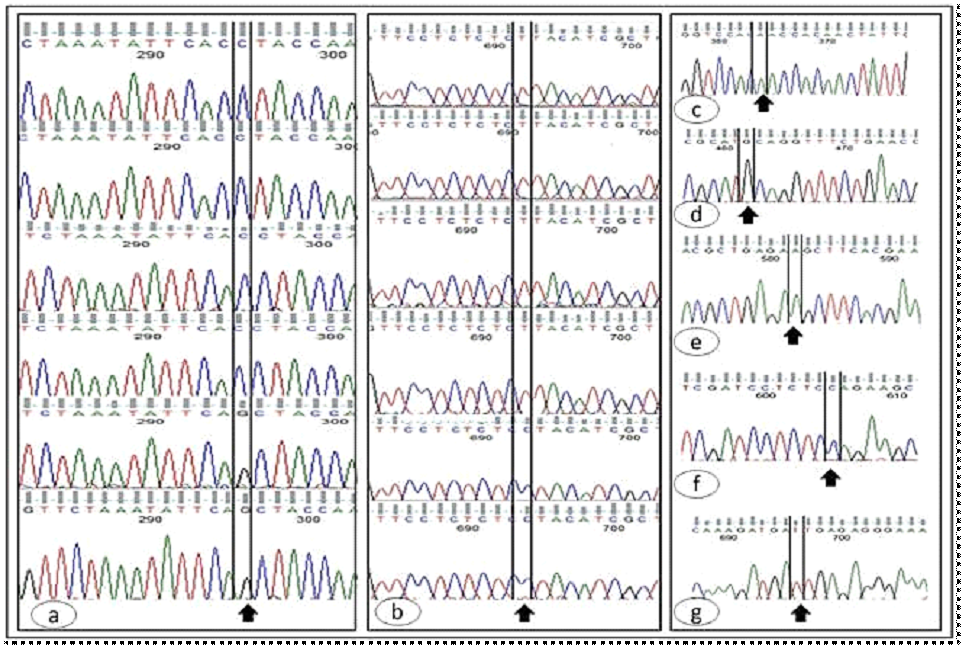

Supplement: S2 Fig — Legend: a) SNP 949th position G>C b) SNP 1346th position C>T c) SNP 1017th position G>A d) Insertion of G between 1114th and 1115th positions e) Insertion A between 1722nd and 1723th f) Insertion C between 1744th and 1745th positions g) Insertion of T between 1835th and 1836th positions. (TIF) [file pone.0151187.s002.tif]

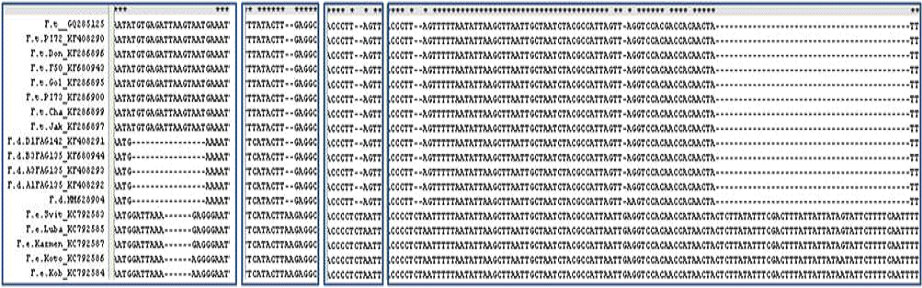

Supplement: S3 Fig — (TIF) [file pone.0151187.s003.tif]
