# Supplementary material for: Genomic Characterization of Phenylalanine Ammonia Lyase Gene in Buckwheat
Source: PLoS One. 2016 Mar 18;11(3):e0151187. doi: 10.1371/journal.pone.0151187 (PMC4798664; doi:10.1371/journal.pone.0151187)
Supplement: S1 Table — Bold letters are significant, P<0.05. (DOCX) [file pone.0151187.s005.docx]

**S1 Table .** Disparity Index (I_D_) test, **1.** Fd_ KF408292, **2.** Fd_KF680944, **3.** Fd_KF408293, **4.** Fd_KF408291, **5.** Fd_HM628904, **6.** Ft_ GQ285125, **7.** Ft_KF680943, **8.** Ft_ FTPI481672, **9.** Ft_KF286897, **10.** Ft_ KF286898, **11.** Ft_ KF286899, **12.** Ft_KF386900, **13.** Ft_KF286896, **14.** Ft_KF286895. Bold letters are significant, P<0.05.

|  | 1 | 2 | 3 | 4 | 5 | 6 | 7 | 8 | 9 | 10 | 11 | 12 | 13 | 14 |
| --- | --- | --- | --- | --- | --- | --- | --- | --- | --- | --- | --- | --- | --- | --- |
| 1 | **0.02** | **0.01** | **0.02** | **0.01** | **0.03** | 0.05 | 0.06 | 0.06 | 0.05 | 0.06 | **0.03** | 0.06 | 0.05 | 0.06 |
| 2 | **0.04** | **0.01** | **0.02** | **0.02** | **0.03** | 0.07 | 0.09 | 0.08 | 0.05 | 0.07 | 0.08 | 0.08 | 0.06 | 0.08 |
| 3 | **0.01** | **0.01** | **0.03** | **0.01** | **0.04** | 0.05 | 0.06 | 0.09 | 0.07 | 0.08 | 0.07 | 0.07 | 0.08 | 0.09 |
| 4 | **0.03** | **0.01** | **0.03** | **0.02** | **0.05** | 0.08 | 0.08 | 0.1 | 0.08 | 0.08 | 0.09 | 0.05 | 0.07 | 0.09 |
| 5 | **0.01** | **0** | **0.02** | **0.03** | **0.03** | 0.05 | 0.05 | **0.06** | **0.03** | **0.04** | 0.05 | **0.03** | **0.05** | **0.04** |
