# Supplementary material for: Genomic Characterization of Phenylalanine Ammonia Lyase Gene in Buckwheat
Source: PLoS One. 2016 Mar 18;11(3):e0151187. doi: 10.1371/journal.pone.0151187 (PMC4798664; doi:10.1371/journal.pone.0151187)
Supplement: S2 Table — (DOCX) [file pone.0151187.s006.docx]

**S2 Table.** Distant Matrix calculated based on Kimura-2-Parameter, **1.** Fd_ KF408292, **2.** Fd_KF680944, **3.** Fd_KF408293, **4.** Fd_KF408291, **5.** Fd_HM628904, **6.** Ft_ GQ285125, **7.** Ft_KF680943, **8.** Ft_ FTPI481672, **9.** Ft_KF286897, **10.** Ft_ KF286898, **11.** Ft_ KF286899, **12.** Ft_KF386900, **13.** Ft_KF286896, **14.** Ft_KF286895, **15.** Fe_KC792586, **16.** Fe_KC792587, **17.** Fe_KC792585, **18.** Fe_KC792583, **19.** Fe_KC792584.

|  | 1 | 2 | 3 | 4 | 5 | 6 | 7 | 8 | 9 | 10 | 11 | 12 | 13 | 14 | 15 | 16 | 17 | 18 | 19 |
| --- | --- | --- | --- | --- | --- | --- | --- | --- | --- | --- | --- | --- | --- | --- | --- | --- | --- | --- | --- |
| 1 |  | 0.00 | 0.00 | 0.00 | 0.01 | 0.01 | 0.01 | 0.01 | 0.01 | 0.01 | 0.01 | 0.01 | 0.01 | 0.01 | 0.02 | 0.01 | 0.01 | 0.01 | 0.02 |
| 2 | 0.01 |  | 0.00 | 0.00 | 0.01 | 0.01 | 0.01 | 0.01 | 0.01 | 0.01 | 0.01 | 0.01 | 0.01 | 0.01 | 0.02 | 0.02 | 0.02 | 0.02 | 0.02 |
| 3 | 0.01 | 0.01 |  | 0.00 | 0.01 | 0.01 | 0.01 | 0.01 | 0.01 | 0.01 | 0.01 | 0.01 | 0.01 | 0.01 | 0.02 | 0.02 | 0.02 | 0.02 | 0.02 |
| 4 | 0.01 | 0.00 | 0.00 |  | 0.01 | 0.01 | 0.01 | 0.01 | 0.01 | 0.01 | 0.01 | 0.01 | 0.01 | 0.01 | 0.02 | 0.02 | 0.02 | 0.02 | 0.02 |
| 5 | 0.02 | 0.02 | 0.03 | 0.03 |  | 0.01 | 0.01 | 0.01 | 0.01 | 0.01 | 0.01 | 0.01 | 0.01 | 0.01 | 0.02 | 0.02 | 0.02 | 0.02 | 0.02 |
| 6 | 0.04 | 0.04 | 0.04 | 0.04 | 0.05 |  | 0.00 | 0.00 | 0.00 | 0.00 | 0.00 | 0.00 | 0.00 | 0.00 | 0.02 | 0.02 | 0.02 | 0.02 | 0.02 |
| 7 | 0.04 | 0.04 | 0.04 | 0.04 | 0.04 | 0.00 |  | 0.00 | 0.00 | 0.00 | 0.00 | 0.00 | 0.00 | 0.00 | 0.02 | 0.01 | 0.01 | 0.01 | 0.02 |
| 8 | 0.04 | 0.04 | 0.04 | 0.04 | 0.04 | 0.00 | 0.00 |  | 0.00 | 0.00 | 0.00 | 0.00 | 0.00 | 0.00 | 0.02 | 0.01 | 0.01 | 0.01 | 0.02 |
| 9 | 0.04 | 0.04 | 0.04 | 0.04 | 0.04 | 0.00 | 0.00 | 0.00 |  | 0.00 | 0.00 | 0.00 | 0.00 | 0.00 | 0.02 | 0.01 | 0.01 | 0.01 | 0.02 |
| 10 | 0.04 | 0.04 | 0.04 | 0.04 | 0.04 | 0.00 | 0.00 | 0.00 | 0.00 |  | 0.00 | 0.00 | 0.00 | 0.00 | 0.02 | 0.01 | 0.01 | 0.01 | 0.02 |
| 11 | 0.04 | 0.04 | 0.04 | 0.04 | 0.04 | 0.00 | 0.00 | 0.00 | 0.00 | 0.00 |  | 0.00 | 0.00 | 0.00 | 0.02 | 0.01 | 0.01 | 0.01 | 0.02 |
| 12 | 0.04 | 0.04 | 0.04 | 0.04 | 0.04 | 0.00 | 0.00 | 0.00 | 0.00 | 0.00 | 0.00 |  | 0.00 | 0.00 | 0.02 | 0.01 | 0.01 | 0.01 | 0.02 |
| 13 | 0.04 | 0.04 | 0.04 | 0.04 | 0.04 | 0.00 | 0.00 | 0.00 | 0.00 | 0.00 | 0.00 | 0.00 |  | 0.00 | 0.02 | 0.01 | 0.01 | 0.01 | 0.02 |
| 14 | 0.04 | 0.04 | 0.04 | 0.04 | 0.04 | 0.00 | 0.00 | 0.00 | 0.00 | 0.00 | 0.00 | 0.00 | 0.00 |  | 0.02 | 0.01 | 0.01 | 0.01 | 0.02 |
| 15 | 0.15 | 0.15 | 0.15 | 0.15 | 0.16 | 0.15 | 0.15 | 0.15 | 0.15 | 0.15 | 0.15 | 0.15 | 0.15 | 0.15 |  | 0.00 | 0.00 | 0.00 | 0.00 |
| 16 | 0.15 | 0.15 | 0.15 | 0.15 | 0.15 | 0.15 | 0.15 | 0.15 | 0.15 | 0.15 | 0.15 | 0.15 | 0.15 | 0.15 | 0.00 |  | 0.00 | 0.00 | 0.00 |
| 17 | 0.15 | 0.15 | 0.15 | 0.15 | 0.15 | 0.15 | 0.15 | 0.15 | 0.15 | 0.15 | 0.15 | 0.15 | 0.15 | 0.15 | 0.00 | 0.00 |  | 0.00 | 0.00 |
| 18 | 0.15 | 0.15 | 0.15 | 0.15 | 0.15 | 0.15 | 0.15 | 0.15 | 0.15 | 0.15 | 0.15 | 0.15 | 0.15 | 0.15 | 0.00 | 0.00 | 0.00 |  | 0.00 |
| 19 | 0.15 | 0.16 | 0.16 | 0.16 | 0.16 | 0.15 | 0.15 | 0.15 | 0.15 | 0.15 | 0.15 | 0.15 | 0.15 | 0.15 | 0.01 | 0.01 | 0.01 | 0.01 |  |
