# Supplementary material for: Genomic Characterization of Phenylalanine Ammonia Lyase Gene in Buckwheat
Source: PLoS One. 2016 Mar 18;11(3):e0151187. doi: 10.1371/journal.pone.0151187 (PMC4798664; doi:10.1371/journal.pone.0151187)
Supplement: S3 Table — Nucleotides represented in capital letters, are the sites in LD and corresponding positions of other species in represented in small letters. D: Linkage Disequilibrium, D’ = Correlation Coefficient of the pairing SNPs, R2 = Coefficient of determination, χ2 = Chi square test, B = Bonferroni corrections, F = Fisher test, **P<0.01, *P<0.05. (DOCX) [file pone.0151187.s007.docx]

**S3 Table.** Linkage Disequilibrium with pair of parsimony informative sites. Nucleotides represented in capital letters, are the sites in LD and corresponding positions of other species in represented in small letters. D: Linkage Disequilibrium, D’= Correlation Coefficient of the pairing SNPs, R^2^ = Coefficient of determination, χ^2^ = Chi square test, B = Bonferroni corrections, F= Fisher test, **P<0.01, *P<0.05.

| Species /Accession |  |  | Sites |  |  |  | D | D’ | R^2^ | χ^2^ | F |
| --- | --- | --- | --- | --- | --- | --- | --- | --- | --- | --- | --- |
|  | 952_1395 | | 822_1061 | | 927_1377 | |  |  |  |  |  |
| *F. tataricum* | |  |  |  |  |  | 0.247 | 1 | 1 | 9.000**B | 0.008* |
| Golden | G | C | a | c | c | - |  |  |  |  |  |
| Donan | G | C | a | c | c | - |  |  |  |  |  |
| FAG50 | G | C | a | c | c | - |  |  |  |  |  |
| Jakuar | C | T | a | c | c | - |  |  |  |  |  |
| PI481673 | C | T | a | c | c | - |  |  |  |  |  |
| GQ285125 | G | C | a | c | c | - |  |  |  |  |  |
| FTPI481672 | G | C | a | c | c | - |  |  |  |  |  |
| Chumoa | C | T | a | c | c | - |  |  |  |  |  |
| Chumey | C | T | a | c | c | - |  |  |  |  |  |
| *F. esculentum* | |  |  |  |  |  | 0.24 | 1 | 1 | 0.02*B | - |
| Luba | c | C | G | C | t | g |  |  |  |  |  |
| Karmen | c | C | G | C | t | g |  |  |  |  |  |
| Svityazyanka | c | C | G | C | t | g |  |  |  |  |  |
| Koto | c | C | A | A | t | a |  |  |  |  |  |
| Koban | c | C | A | A | t | a |  |  |  |  |  |
| *F. dibotrys* | |  |  |  |  |  | 0.24 | 1 | 1 | 0.02* | - |
| FDD1FAG142 | c | C | - | - | T | C |  |  |  |  |  |
| FCA1FAG135 | c | C | - | - | C | T |  |  |  |  |  |
| FCA3FAG135 | c | C | - | - | T | C |  |  |  |  |  |
| FCB3FAG135 | c | C | - | - | T | C |  |  |  |  |  |
| HM628904 |  |  |  |  | C | T |  |  |  |  |  |
