# Supplementary material for: Genomic Characterization of Phenylalanine Ammonia Lyase Gene in Buckwheat
Source: PLoS One. 2016 Mar 18;11(3):e0151187. doi: 10.1371/journal.pone.0151187 (PMC4798664; doi:10.1371/journal.pone.0151187)
Supplement: S4 Table — (DOCX) [file pone.0151187.s008.docx]

**S4 Table.** Primers utilised for PCR amplification and sequencing

| S.No | Primer Name | Sequence 5’ to 3’ | Tm ^o^C |
| --- | --- | --- | --- |
| 1 | FT E1 Start F | A T G G G G G T C T C A A A C G G A | 65.4 |
| 2 | FT E2 Nr R | T C G C C A G A A G C A G T G A T G | 64.1 |
| 3 | FT UTR Start F | C C C C A T A A T G G T C C A A C G | 63.6 |
| 4 | FT E1 Fr R | G T C C T A C G A T G A G A A G T A G C T C C | 62.1 |
| 5 | FT E1 VFr F | T A A G G A A G G C G G T G C T C T T | 63.8 |
| 6 | FT E2 AMed R | G C T T C T G A G A G G A T C G A G T T C | 62.1 |
| 7 | FT E2 BMed F | G A G C T T C A C G A A C T C G A T C C | 63.9 |
| 8 | FT E2 End R | C T A G C A G A T A G G C A G A G G A G C A | 64.8 |
| 9 | FT E2 Start F | G T T T C T G A A C G C C G G A G T A T | 62.9 |
| 10 | FE E1 F | GTCGAGCTGGATGAGGAGG | 65.1 |
| 11 | FE E2 R | CTCCAGTGAGGGCAGTGAA | 64.3 |
