# Supplementary material for: Genomic Characterization of Phenylalanine Ammonia Lyase Gene in Buckwheat
Source: PLoS One. 2016 Mar 18;11(3):e0151187. doi: 10.1371/journal.pone.0151187 (PMC4798664; doi:10.1371/journal.pone.0151187)
Supplement: S5 Table — (DOCX) [file pone.0151187.s009.docx]

**S5 Table.** PCR master mix concentration with Tetra primers adopted for SNP allele validation.

| Reagents | Conc. | Quantity µl | Conc./25µl | Numbers | Answer µl | Final Conc. |
| --- | --- | --- | --- | --- | --- | --- |
| dNTP | 1µl = 2.5 mM | 2 | 5000 µM | 96.2 | 192.4 | 200 µM/µl |
| Taq | 1µl = 5 Unit | 0.3 | 1.5U | 96.2 | 28.86 | 0.06 U/µl |
| 10X Buffer | 5µl = 20X | 2.5 | 10X | 96.2 | 240.5 | 0.4X/µl |
| 50mM MgCl2 | 1µl = 50 mM | 1.5 | 75 mM | 96.2 | 144.3 | 3 mM/µl |
| FOP | 1µl = 10µM | 0.5 | 5 µM | 96.2 | 48.1 | 0.2µM/µl |
| ROP | 1µl = 10µM | 0.5 | 5 µM | 96.2 | 48.1 | 0.2 µM/µl |
| FIP | 1µl = 10 µM | 1 | 10 µM | 96.2 | 96.2 | 0.4 µM/µl |
| RIP | 1µl = 10 µM | 1 | 10 µM | 96.2 | 96.2 | 0.4 µM/µl |
| DNA | 1µl = 50 ng | 4 | 200 ng | 96.2 | n/a | 8 ng/µl |
| Water | 1µl = 55.50µM | 11.7 | 649.44 µM | 96.2 | 1125.54 | 25.97 µM/µl |
| Total MM=2020.2µl Each MM=21µl DNA =4µl Total =25µl | | | | | |  |
