# Supplementary material for: Genomic Characterization of Phenylalanine Ammonia Lyase Gene in Buckwheat
Source: PLoS One. 2016 Mar 18;11(3):e0151187. doi: 10.1371/journal.pone.0151187 (PMC4798664; doi:10.1371/journal.pone.0151187)
Supplement: S6 Table — (DOCX) [file pone.0151187.s010.docx]

**S6 Table.** Tetra primers of SNP position 949.

| Primer Name | Primer 5’-3’ | Tm ^o^C | GC% | Secondary Structure |
| --- | --- | --- | --- | --- |
| FIP-949G | GCCGTCATTTGAGTTCTAAATATTGAG | 65.4 | 37 | Very weak |
| RIP-949C | TATTAAAAACTAAGGGTTGGTTGGTTGG | 66.6 | 35.7 | Very Weak |
| FOP-949 | TGATCGATTTTGGGTAGTAATTAATGGA | 66.5 | 32.1 | None |
| ROP-949 | GAAGAGTGTTGATCCGTACAAGCATAG | 66.6 | 44.4 | Very Weak |
